# Editorial: Making Science Fun – A Tribute to Our Colleague and Friend, Prof. Antonius G. Rolink (1953–2017)

**DOI:** 10.3389/fimmu.2018.02915

**Published:** 2018-12-19

**Authors:** Hermann Eibel, Thomas Winkler, Rhodri Ceredig

**Affiliations:** ^1^Center for Chronic Immunodeficiency, University Medical Center Freiburg, Freiburg, Germany; ^2^Nikolaus-Fiebiger-Zentrum für Molekulare Medizin, Universität Erlangen-Nürnberg, Erlangen, Germany; ^3^Discipline of Physiology, College of Medicine and Nursing Health Science, National University of Ireland, Galway, Ireland

**Keywords:** lymphocyte development and function, B cells, T cells, hemopoiesis, graft versus host desease, BAFF - B-cell activating factor, treg cells, monoclonal antibodies

## Research Topic Content

This Research Topic was organized to honor the memory of our dear friend Antonius “Ton” Rolink (April 19, 1953–August 06, 2017). The contributions are from his former students, colleagues, and collaborators. In the form of original research and review articles, these papers cover many of Ton's scientific interests in different aspects of lymphocyte development in mouse and man. Thus, the majority of articles concern B cell biology, ranging from papers by Pang et al. and Kim and Schaniel on stem cells to Klein et al. and Winkler and Mårtensson on B cell precursors, Brennecke et al. and Hobeika et al. on inducible B cell development, Smulski and Eibel and Kowalczyk-Quintans et al. on B cell Activating Factor (BAFF) and the impact of BIM on B cell survival, Greaves et al. on tolerance and Song and Matthias on the formation of germinal centers. However, Ton's research was also motivated by his continuous interest in T cells and graft-versus-host reactions, lymphoid tumours and in the application of antibody technology to develop novel therapeutic approaches. These subjects are covered by the contributions of Mori and Pieters on T cells, Calvo-Asensio et al. on T cell progenitors, by the article of Ghia on leukemia development, by Heiler et al. on GvH and by Hellmann et al. on human antibody libraries.

## Ton's Scientific Career

Ton Rolink began his scientific career as a PhD student in the group of Ernst Gleichman at the University of Amsterdam focussing on the mechanisms of T cell mediated immunopathology during Graft versus Host Disease (GvHD). This resulted in a remarkable output of 12 publications, including five in the Journal of Experimental Medicine (1–12).

In 1983, he moved to the Basel Institute of Immunology (BII) as a Scientific Member, joining the laboratory of Fritz Melchers. In the following years, the team developed the technologies and skills that led to the discovery of fundamental principles in B cell development (13–16), B cell tolerance (17) and autoimmunity (18–20). Over the years, Ton and his collaborators generated many monoclonal antibodies, some of which, including those to precursor B cells (21), the IL-5 receptor (22), CD40 (23), CD93 (24), and BAFF (25), resulted in numerous novel findings and publications.

One of the key technical advances in which Ton made a significant contribution was the establishment of stroma cell-based *in vitro* system allowing the cultivation of B cell precursors starting with single hematopoietic stem cells (26). Since Ton indeed had “green fingers” for growing cells, he was naturally gifted at cell culture. Therefore, when Stephen Nutt in Busslinger's laboratory showed that the transcription factor Pax5 (or BSAP) was essential for B lymphopoiesis (27) the scientific collaboration established with Ton's laboratory continued. This culminated in 1999 with two seminal articles in Nature describing how Pax5-deficient pro B cell lines could proceed along different developmental pathways to become antigen-presenting dendritic cells, osteoclasts, granulocytes or natural killer cells *in vitro* and to T cells following reconstitution of mice *in vivo* (28, 29). The realization that the transcription factor Pax5 was a master regulator of B cell development had a profound influence on the field of haematopoiesis and lymphopoiesis and opened new research avenues allowing in depth analysis of the roles of other transcription factors in the regulation of lymphocyte development (30–36).

By refining the conditions of *in vitro* B cell development, the roles of different chemokines and cytokines implicated in B cell development and homeostasis were also investigated (37–41) including the detailed dissection in mouse and man of the role of the B cell Activating Factor (BAFF) (42, 43) in normal B cell homeostasis and as well as in the development of autoimmunity (44–47). Using emerging molecular technologies, Ton and his group dissected B cell development at the single cell level, analysing their genotypic and transcriptomic profiles (48–50). For this, techniques capable of identifying rearrangement of D_H_ and J_H_ genes on one immunoglobulin heavy chain allele, corresponding to one molecule of rearranged DNA, could be detected (51). Without a doubt, Ton's research contributions were recognized world-wide and he became one of the leaders in studies of mouse and human B cell development.

Having helped to show the multi-lineage differentiation capacity of B220^+^CD117^low^CD19^−^ Pax 5 KO pro-B cell lines, Ton became interested in trying to identify a cell type with identical phenotype and equivalent differentiation capacity in the bone marrow of normal mice. This progenitor was indeed identified and referred to as an Early Progenitor with Lymphocyte and Myeloid Potential, or EPLM (52). More recent experiments using additional markers and taking advantage of single cell RNA sequencing, has revealed that the original EPLM population is both phenotypically and genotypically heterogenous with the earliest member being already committed to either lymphoid or myeloid lineages (53, 54).

Seeing that mice could be reconstituted with genetically-modified B cell progenitors grown *in vitro* and that Pax5 KO (29) and EPLM (52) could reconstitute the T cell compartment led Ton to expand his studies to T cell development (55). Realising that therapeutic use of progenitors grown on stromal cells would pose difficulties for clinical approval, he established stromal cell-free culture conditions whereby mouse and human T cell progenitors could be expanded and differentiated without stromal cells. The system developed was affectionately called “the plastic thymus” (56) allowing the dissection of signals involved in T cell commitment (57–59). In recent years, this led to a series of experiments investigating the possible instructive role of cytokines, in particular IL-7 and Flt3L, on lymphocyte development (53, 60–62).

This T cell work set the foundation for Ton's subsequent studies on T cell autoimmunity. Using T cell progenitors from genetically-modified mice, his group was able to expand and reconstitute the T cell compartment of immunodeficient recipients (63). However, the “plastic thymus” could not replace one of the key functions of the thymus, namely the generation of regulatory T cells (64). Without endogenous regulatory T cells, after a few weeks recipient mice reconstituted with *in vitro* generated T cell progenitors invariably developed immunopathology reminiscent of that seen in GvHD. Addition of regulatory T cells to the T progenitor inoculum was sufficient to prevent disease onset (64), providing Ton with a functional *in vivo* assay for regulatory T cell function.

Ton's interest in autoimmunity associated with GvHD, initiated whilst a PhD student and continued at BII and the University of Basel, interested him until the very end of his career. In his latest publications, he looked at this topic from a T-cell (Heiler et al.) and a B-cell perspective (65). In the first, Heiler et al. analyzed the contribution of the different T cell compartments at various stages of disease, the latter (65) showed the processes by which self-reactive antibodies might arise in aged mice.

**Figure 1:**
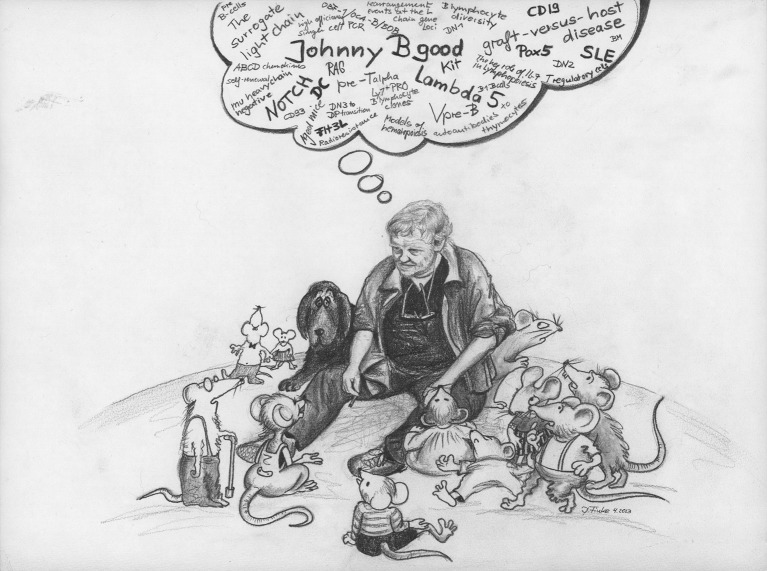
The cartoon has been painted and kindly provided by Professor Daniela Finke, who has been for many years Ton Rolink's colleague at the Department of Biomedicine, University of Basel.

Ton Rolink's key findings in the field of lymphocyte biology as well as his talent for developing numerous monoclonal antibodies and cell culture systems now used as research tools in many laboratories world-wide, were always complemented by his open, friendly, and generous personality and his infectious good humor. Throughout his scientific career, Ton worked almost daily at the bench. He was always ready to share his ideas, tools and expertise with his colleagues and collaborators, which in times of a steadily growing number of Material Transfer Agreements (MTAs), research contracts and “highly confidential information,” was a rare phenomenon among scientists. Therefore, Ton will not only be remembered for his exceptional work ethic and output, but also, and maybe even more, for his exceptional way of making science fun. He will also be remembered by his colleagues and collaborators through their projects that live and thrive thanks to his reagents and spirit (Figure 1).

His complete bibliography can be found in the Appendix and the following is a link to Ton's publications on PubMed: https://www.ncbi.nlm.nih.gov/pubmed/?term=Rolink+A.

## Author Contributions

All authors listed have made a substantial, direct and intellectual contribution to the work, and approved it for publication.

### Conflict of Interest Statement

The authors declare that the research was conducted in the absence of any commercial or financial relationships that could be construed as a potential conflict of interest.

## Appendix

### Ton's Complete Bibliography

1. Van der Veen F, Rolink AG, Gleichmann E. Diseases caused by reactions of T lymphocytes to incompatible structures of the major histocompatibility complex. IV. Autoantibodies to nuclear antigens. Clin Exp Immunol. 1981;46(3):589-96.
2. van der Veen JP, Rolink AG, Gleichmann E. Diseases caused by reactions of T lymphocytes to incompatible structures of the major histocompatibility complex. III. Autoantibodies to thymocytes. J Immunol. 1981;127(4):1281-6.
3. Van Elven EH, Rolink AG, Veen FV, Gleichmann E. Capacity of genetically different T lymphocytes to induce lethal graft-versus-host disease correlates with their capacity to generate suppression but not with their capacity to generate anti-F1 killer cells. A non-H-2 locus determines the inability to induce lethal graft-versus-host disease. J Exp Med. 1981;153(6):1474-88.
4. van Elven EH, van der Veen FM, Rolink AG, Issa P, Duin TM, Gleichmann E. Diseases caused by reactions of T lymphocytes to incompatible structures of the major histocompatibility complex. V. High titers of IgG autoantibodies to double-stranded DNA. J Immunol. 1981;127(6):2435-8.
5. Rolink AG, Radaszkiewicz T, Pals ST, van der Meer WG, Gleichmann E. Allosuppressor and allohelper T cells in acute and chronic graft-vs-host disease. I. Alloreactive suppressor cells rather than killer T cells appear to be the decisive effector cells in lethal graft-vs.-host disease. J Exp Med. 1982;155(5):1501-22.
6. van der Veen FM, Rolink AG, Gleichmann E. Autoimmune disease strongly resembling systemic lupus erythematosus (SLE) in F1 mice undergoing graft-versus-host reaction (GVHR). Adv Exp Med Biol. 1982;149:669-77.
7. van Rappard-van der Veen FM, Rolink AG, Gleichmann E. Diseases caused by reactions of T lymphocytes towards incompatible structures of the major histocompatibility complex. VI. Autoantibodies characteristic of systemic lupus erythematosus induced by abnormal T-B cell cooperation across I-E. J Exp Med. 1982;155(5):1555-60.
8. Rolink AG, Gleichmann E. Allosuppressor- and allohelper-T cells in acute and chronic graft-vs.-host (GVH) disease. III. Different Lyt subsets of donor T cells induce different pathological syndromes. J Exp Med. 1983;158(2):546-58.
9. Rolink AG, Gleichmann H, Gleichmann E. Diseases caused by reactions of T lymphocytes to incompatible structures of the major histocompatibility complex. VII. Immune-complex glomerulonephritis. J Immunol. 1983;130(1):209-15.
10. Rolink AG, Pals ST, Gleichmann E. Allosuppressor and allohelper T cells in acute and chronic graft-vs.-host disease. II. F1 recipients carrying mutations at H-2K and/or I-A. J Exp Med. 1983;157(2):755-71.
11. Rolink AG, Van der Meer W, Melief CJ, Gleichmann E. Intra-H-2 and T cell requirements for the induction of maximal positive and negative allogeneic effects in vitro. Eur J Immunol. 1983;13(3):191-7.
12. Gleichmann E, Pals ST, Rolink AG, Radaszkiewicz T, Gleichmann H. Graft-versus-host reactions: clues to the etiopathology of a spectrum of immunological diseases. Immunol Today. 1984;5(11):324-32.
13. Lernhardt W, Karasuyama H, Rolink A, Melchers F. Control of the cell cycle of murine B lymphocytes: the nature of alpha- and beta-B-cell growth factors and of B-cell maturation factors. Immunol Rev. 1987;99:241-62.
14. Palacios R, Karasuyama H, Rolink A. Ly1+ PRO-B lymphocyte clones. Phenotype, growth requirements and differentiation in vitro and in vivo. EMBO J. 1987;6(12):3687-93.
15. Rolink AG, Radaszkiewicz T, Melchers F. The autoantigen-binding B cell repertoires of normal and of chronically graft-versus-host-diseased mice. J Exp Med. 1987;165(6):1675-87.
16. Karasuyama H, Rolink A, Melchers F. Recombinant interleukin 2 or 5, but not 3 or 4, induces maturation of resting mouse B lymphocytes and propagates proliferation of activated B cell blasts. J Exp Med. 1988;167(4):1377-90.
17. Rolink AG, Radaszkiewicz T, Melchers F. Monoclonal autoantibodies specific for kidney proximal tubular brush border from mice with experimentally induced chronic graft-versus-host disease. Scand J Immunol. 1988;28(1):29-41.
18. Rolink AG, Thalmann P, Berger C, Radaszkiewicz T, Melchers F. Autoreactive B-cell repertoire in mice with chronic graft versus host disease. Mol Immunol. 1988;25(11):1217-22.
19. Melchers F, Bauer SR, Berger C, Karasuyama H, Kudo A, Rolink A, et al. Precursor B lymphocytes–specific monoclonal antibodies and genes. Adv Exp Med Biol. 1989;254:87-93.
20. Melchers F, Strasser A, Bauer SR, Kudo A, Thalmann P, Rolink A. Cellular stages and molecular steps of murine B-cell development. Cold Spring Harb Symp Quant Biol. 1989;54 Pt 1:183-9.
21. Palacios R, Stuber S, Rolink A. The epigenetic influences of bone marrow and fetal liver stroma cells on the developmental potential of Ly-1+ pro-B lymphocyte clones. Eur J Immunol. 1989;19(2):347-56.
22. Rolink AG, Melchers F, Palacios R. Monoclonal antibodies reactive with the mouse interleukin 5 receptor. J Exp Med. 1989;169(5):1693-701.
23. Strasser A, Rolink A, Melchers F. One synchronous wave of B cell development in mouse fetal liver changes at day 16 of gestation from dependence to independence of a stromal cell environment. J Exp Med. 1989;170(6):1973-86.
24. Devos R, Tavernier J, Plaetinck G, Van der Heyden J, Rolink A, Fiers W. Expression of the murine interleukin-5 receptor on Xenopus laevis oocytes. Biochem Biophys Res Commun. 1990;172(2):570-5.
25. Rolink AG, Thalmann P, Kikuchi Y, Erdei A. Characterization of the interleukin 5-reactive splenic B cell population. Eur J Immunol. 1990;20(9):1949-56.
26. Tary-Lehmann M, Rolink AG, Lehmann PV, Nagy ZA, Hurtenbach U. Induction of graft versus host-associated immunodeficiency by CD4+ T cell clones. J Immunol. 1990;145(7):2092-8.
27. Devos R, Vandekerckhove J, Rolink A, Plaetinck G, Van der Heyden J, Fiers W, et al. Amino acid sequence analysis of a mouse interleukin 5 receptor protein reveals homology with a mouse interleukin 3 receptor protein. Eur J Immunol. 1991;21(5):1315-7.
28. Melchers F, Strasser A, Bauer SR, Kudo A, Thalmann P, Rolink A. B cell development in fetal liver. Adv Exp Med Biol. 1991;292:201-5.
29. Mita S, Takaki S, Hitoshi Y, Rolink AG, Tominaga A, Yamaguchi N, et al. Molecular characterization of the beta chain of the murine interleukin 5 receptor. Int Immunol. 1991;3(7):665-72.
30. Rolink A, Kudo A, Karasuyama H, Kikuchi Y, Melchers F. Long-term proliferating early pre B cell lines and clones with the potential to develop to surface Ig-positive, mitogen reactive B cells in vitro and in vivo. EMBO J. 1991;10(2):327-36.
31. Rolink A, Kudo A, Melchers F. Long-term proliferating early pre-B-cell lines and clones with the potential to develop to surface immunoglobulin-positive, mitogen-reactive B-cells in vitro and in vivo. Biochem Soc Trans. 1991;19(2):275-6.
32. Rolink A, Melchers F. Molecular and cellular origins of B lymphocyte diversity. Cell. 1991;66(6):1081-94.
33. Rolink A, Streb M, Melchers F. The kappa/lambda ratio in surface immunoglobulin molecules on B lymphocytes differentiating from DHJH-rearranged murine pre-B cell clones in vitro. Eur J Immunol. 1991;21(11):2895-8.
34. Rolink A, Streb M, Nishikawa S, Melchers F. The c-kit-encoded tyrosine kinase regulates the proliferation of early pre-B cells. Eur J Immunol. 1991;21(10):2609-12.
35. Kudo A, Thalmann P, Sakaguchi N, Davidson WF, Pierce JH, Kearney JF, et al. The expression of the mouse VpreB/lambda 5 locus in transformed cell lines and tumors of the B lineage differentiation pathway. Int Immunol. 1992;4(8):831-40.
36. Melchers F, Haasner D, Karasuyama H, Reininger L, Rolink A. Progenitor and precursor B lymphocytes of mice. Proliferation and differentiation in vitro and population, differentiation and turnover in SCID mice in vivo of normal and abnormal cells. Curr Top Microbiol Immunol. 1992;182:3-12.
37. Melchers F, Haasner D, Streb M, Rolink A. B-lymphocyte lineage-committed, IL-7 and stroma cell-reactive progenitors and precursors, and their differentiation to B cells. Adv Exp Med Biol. 1992;323:111-7.
38. Oltz EM, Yancopoulos GD, Morrow MA, Rolink A, Lee G, Wong F, et al. A novel regulatory myosin light chain gene distinguishes pre-B cell subsets and is IL-7 inducible. EMBO J. 1992;11(7):2759-67.
39. Reininger L, Radaszkiewicz T, Kosco M, Melchers F, Rolink AG. Development of autoimmune disease in SCID mice populated with long-term "in vitro" proliferating (NZB x NZW)F1 pre-B cells. J Exp Med. 1992;176(5):1343-53.
40. Grawunder U, Haasner D, Melchers F, Rolink A. Rearrangement and expression of kappa light chain genes can occur without mu heavy chain expression during differentiation of pre-B cells. Int Immunol. 1993;5(12):1609-18.
41. Grawunder U, Melchers F, Rolink A. Interferon-gamma arrests proliferation and causes apoptosis in stromal cell/interleukin-7-dependent normal murine pre-B cell lines and clones in vitro, but does not induce differentiation to surface immunoglobulin-positive B cells. Eur J Immunol. 1993;23(2):544-51.
42. Karasuyama H, Rolink A, Melchers F. A complex of glycoproteins is associated with VpreB/lambda 5 surrogate light chain on the surface of mu heavy chain-negative early precursor B cell lines. J Exp Med. 1993;178(2):469-78.
43. Melchers F, Karasuyama H, Haasner D, Bauer S, Kudo A, Sakaguchi N, et al. The surrogate light chain in B-cell development. Immunol Today. 1993;14(2):60-8.
44. Rolink A, Grawunder U, Haasner D, Strasser A, Melchers F. Immature surface Ig+ B cells can continue to rearrange kappa and lambda L chain gene loci. J Exp Med. 1993;178(4):1263-70.
45. Rolink A, Haasner D, Nishikawa S, Melchers F. Changes in frequencies of clonable pre B cells during life in different lymphoid organs of mice. Blood. 1993;81(9):2290-300.
46. Rolink A, Karasuyama H, Grawunder U, Haasner D, Kudo A, Melchers F. B cell development in mice with a defective lambda 5 gene. Eur J Immunol. 1993;23(6):1284-8.
47. Rolink A, Melchers F. Generation and regeneration of cells of the B-lymphocyte lineage. Curr Opin Immunol. 1993;5(2):207-17.
48. Rolink A, Melchers F. B lymphopoiesis in the mouse. Adv Immunol. 1993;53:123-56.
49. Haasner D, Rolink A, Melchers F. Influence of surrogate L chain on DHJH-reading frame 2 suppression in mouse precursor B cells. Int Immunol. 1994;6(1):21-30.
50. Karasuyama H, Rolink A, Shinkai Y, Young F, Alt FW, Melchers F. The expression of Vpre-B/lambda 5 surrogate light chain in early bone marrow precursor B cells of normal and B cell-deficient mutant mice. Cell. 1994;77(1):133-43.
51. Kirberg J, Baron A, Jakob S, Rolink A, Karjalainen K, von Boehmer H. Thymic selection of CD8+ single positive cells with a class II major histocompatibility complex-restricted receptor. J Exp Med. 1994;180(1):25-34.
52. Melchers F, Haasner D, Grawunder U, Kalberer C, Karasuyama H, Winkler T, et al. Roles of IgH and L chains and of surrogate H and L chains in the development of cells of the B lymphocyte lineage. Annu Rev Immunol. 1994;12:209-25.
53. Rolink A, Grawunder U, Winkler TH, Karasuyama H, Melchers F. IL-2 receptor alpha chain (CD25, TAC) expression defines a crucial stage in pre-B cell development. Int Immunol. 1994;6(8):1257-64.
54. Rolink A, Karasuyama H, Haasner D, Grawunder U, Martensson IL, Kudo A, et al. Two pathways of B-lymphocyte development in mouse bone marrow and the roles of surrogate L chain in this development. Immunol Rev. 1994;137:185-201.
55. Rolink AG, Reininger L, Oka Y, Kalberer CP, Winkler TH, Melchers F. Repopulation of SCID mice with long-term in vitro proliferating pre-B-cell lines from normal and autoimmune disease-prone mice. Res Immunol. 1994;145(5):353-6.
56. Young F, Ardman B, Shinkai Y, Lansford R, Blackwell TK, Mendelsohn M, et al. Influence of immunoglobulin heavy- and light-chain expression on B-cell differentiation. Genes Dev. 1994;8(9):1043-57.
57. Andersson J, Melchers F, Rolink A. Stimulation by T cell independent antigens can relieve the arrest of differentiation of immature auto-reactive B cells in the bone marrow. Scand J Immunol. 1995;42(1):21-33.
58. Bruno L, Rocha B, Rolink A, von Boehmer H, Rodewald HR. Intra- and extra-thymic expression of the pre-T cell receptor alpha gene. Eur J Immunol. 1995;25(7):1877-82.
59. Ghia P, Gratwohl A, Signer E, Winkler TH, Melchers F, Rolink AG. Immature B cells from human and mouse bone marrow can change their surface light chain expression. Eur J Immunol. 1995;25(11):3108-14.
60. Grawunder U, Leu TM, Schatz DG, Werner A, Rolink AG, Melchers F, et al. Down-regulation of RAG1 and RAG2 gene expression in preB cells after functional immunoglobulin heavy chain rearrangement. Immunity. 1995;3(5):601-8.
61. Grawunder U, Rolink A, Melchers F. Induction of sterile transcription from the kappa L chain gene locus in V(D)J recombinase-deficient progenitor B cells. Int Immunol. 1995;7(12):1915-25.
62. Jessberger R, Riwar B, Rolink A, Rodewald HR. Stimulation of defective DNA transfer activity in recombination deficient SCID cell extracts by a 72-kDa protein from wild-type thymocytes. J Biol Chem. 1995;270(12):6788-97.
63. Melchers F, Rolink A, Grawunder U, Winkler TH, Karasuyama H, Ghia P, et al. Positive and negative selection events during B lymphopoiesis. Curr Opin Immunol. 1995;7(2):214-27.
64. Oka Y, Rolink AG, Suematsu S, Kishimoto T, Melchers F. An interleukin-6 transgene expressed in B lymphocyte lineage cells overcomes the T cell-dependent establishment of normal levels of switched immunoglobulin isotypes. Eur J Immunol. 1995;25(5):1332-7.
65. Rolink A, Ghia P, Grawunder U, Haasner D, Karasuyama H, Kalberer C, et al. In-vitro analyses of mechanisms of B-cell development. Semin Immunol. 1995;7(3):155-67.
66. ten Boekel E, Melchers F, Rolink A. The status of Ig loci rearrangements in single cells from different stages of B cell development. Int Immunol. 1995;7(6):1013-9.
67. Winkler TH, Melchers F, Rolink AG. Interleukin-3 and interleukin-7 are alternative growth factors for the same B-cell precursors in the mouse. Blood. 1995;85(8):2045-51.
68. Winkler TH, Rolink A, Melchers F, Karasuyama H. Precursor B cells of mouse bone marrow express two different complexes with the surrogate light chain on the surface. Eur J Immunol. 1995;25(2):446-50.
69. Ghia P, ten Boekel E, Sanz E, de la Hera A, Rolink A, Melchers F. Ordering of human bone marrow B lymphocyte precursors by single-cell polymerase chain reaction analyses of the rearrangement status of the immunoglobulin H and L chain gene loci. J Exp Med. 1996;184(6):2217-29.
70. Grawunder U, Schatz DG, Leu TM, Rolink A, Melchers F. The half-life of RAG-1 protein in precursor B cells is increased in the absence of RAG-2 expression. J Exp Med. 1996;183(4):1731-7.
71. Karasuyama H, Rolink A, Melchers F. Surrogate light chain in B cell development. Adv Immunol. 1996;63:1-41.
72. Mertsching E, Grawunder U, Meyer V, Rolink T, Ceredig R. Phenotypic and functional analysis of B lymphopoiesis in interleukin-7-transgenic mice: expansion of pro/pre-B cell number and persistence of B lymphocyte development in lymph nodes and spleen. Eur J Immunol. 1996;26(1):28-33.
73. Oka Y, Rolink AG, Andersson J, Kamanaka M, Uchida J, Yasui T, et al. Profound reduction of mature B cell numbers, reactivities and serum Ig levels in mice which simultaneously carry the XID and CD40 deficiency genes. Int Immunol. 1996;8(11):1675-85.
74. Reininger L, Winkler TH, Kalberer CP, Jourdan M, Melchers F, Rolink AG. Intrinsic B cell defects in NZB and NZW mice contribute to systemic lupus erythematosus in (NZB x NZW)F1 mice. J Exp Med. 1996;184(3):853-61.
75. Rolink A, Haasner D, Melchers F, Andersson J. The surrogate light chain in mouse B-cell development. Int Rev Immunol. 1996;13(4):341-56.
76. Rolink A, Melchers F. B-cell development in the mouse. Immunol Lett. 1996;54(2-3):157-61.
77. Rolink A, Melchers F, Andersson J. The SCID but not the RAG-2 gene product is required for S mu-S epsilon heavy chain class switching. Immunity. 1996;5(4):319-30.
78. Rolink A, ten Boekel E, Melchers F, Fearon DT, Krop I, Andersson J. A subpopulation of B220+ cells in murine bone marrow does not express CD19 and contains natural killer cell progenitors. J Exp Med. 1996;183(1):187-94.
79. Schubart DB, Rolink A, Kosco-Vilbois MH, Botteri F, Matthias P. B-cell-specific coactivator OBF-1/OCA-B/Bob1 required for immune response and germinal centre formation. Nature. 1996;383(6600):538-42.
80. Cavelier P, Nato F, Coquilleau I, Rolink A, Rougeon F, Goodhardt M. B lineage-restricted rearrangement of a human Ig kappa transgene. Eur J Immunol. 1997;27(7):1626-31.
81. D'Apuzzo M, Rolink A, Loetscher M, Hoxie JA, Clark-Lewis I, Melchers F, et al. The chemokine SDF-1, stromal cell-derived factor 1, attracts early stage B cell precursors via the chemokine receptor CXCR4. Eur J Immunol. 1997;27(7):1788-93.
82. Kalberer CP, Reininger L, Melchers F, Rolink AG. Priming of helper T cell-dependent antibody responses by hemagglutinin-transgenic B cells. Eur J Immunol. 1997;27(9):2400-7.
83. Melamed D, Kench JA, Grabstein K, Rolink A, Nemazee D. A functional B cell receptor transgene allows efficient IL-7-independent maturation of B cell precursors. J Immunol. 1997;159(3):1233-9.
84. Nutt SL, Urbanek P, Rolink A, Busslinger M. Essential functions of Pax5 (BSAP) in pro-B cell development: difference between fetal and adult B lymphopoiesis and reduced V-to-DJ recombination at the IgH locus. Genes Dev. 1997;11(4):476-91.
85. Siwarski D, Muller U, Andersson J, Notario V, Melchers F, Rolink A, et al. Structure and expression of the c-Myc/Pvt 1 megagene locus. Curr Top Microbiol Immunol. 1997;224:67-72.
86. ten Boekel E, Melchers F, Rolink AG. Changes in the V(H) gene repertoire of developing precursor B lymphocytes in mouse bone marrow mediated by the pre-B cell receptor. Immunity. 1997;7(3):357-68.
87. Ceredig R, ten Boekel E, Rolink A, Melchers F, Andersson J. Fetal liver organ cultures allow the proliferative expansion of pre-B receptor-expressing pre-B-II cells and the differentiation of immature and mature B cells in vitro. Int Immunol. 1998;10(1):49-59.
88. Colonna M, Samaridis J, Cella M, Angman L, Allen RL, O'Callaghan CA, et al. Human myelomonocytic cells express an inhibitory receptor for classical and nonclassical MHC class I molecules. J Immunol. 1998;160(7):3096-100.
89. Dinkel A, Warnatz K, Ledermann B, Rolink A, Zipfel PF, Burki K, et al. The transcription factor early growth response 1 (Egr-1) advances differentiation of pre-B and immature B cells. J Exp Med. 1998;188(12):2215-24.
90. Engel H, Bogen B, Muller U, Andersson J, Rolink A, Weiss S. Expression level of a transgenic lambda2 chain results in isotype exclusion and commitment to B1 cells. Eur J Immunol. 1998;28(8):2289-99.
91. Ghia P, ten Boekel E, Rolink AG, Melchers F. B-cell development: a comparison between mouse and man. Immunol Today. 1998;19(10):480-5.
92. Gunthert U, Schwarzler C, Wittig B, Laman J, Ruiz P, Stauder R, et al. Functional involvement of CD44, a family of cell adhesion molecules, in immune responses, tumour progression and haematopoiesis. Adv Exp Med Biol. 1998;451:43-9.
93. Kistler B, Rolink A, Marienfeld R, Neumann M, Wirth T. Induction of nuclear factor-kappa B during primary B cell differentiation. J Immunol. 1998;160(5):2308-17.
94. Morrison AM, Nutt SL, Thevenin C, Rolink A, Busslinger M. Loss- and gain-of-function mutations reveal an important role of BSAP (Pax-5) at the start and end of B cell differentiation. Semin Immunol. 1998;10(2):133-42.
95. Nutt SL, Morrison AM, Dorfler P, Rolink A, Busslinger M. Identification of BSAP (Pax-5) target genes in early B-cell development by loss- and gain-of-function experiments. EMBO J. 1998;17(8):2319-33.
96. Osmond DG, Rolink A, Melchers F. Murine B lymphopoiesis: towards a unified model. Immunol Today. 1998;19(2):65-8.
97. Rijkers T, Van Den Ouweland J, Morolli B, Rolink AG, Baarends WM, Van Sloun PP, et al. Targeted inactivation of mouse RAD52 reduces homologous recombination but not resistance to ionizing radiation. Mol Cell Biol. 1998;18(11):6423-9.
98. Rolink AG, Andersson J, Melchers F. Characterization of immature B cells by a novel monoclonal antibody, by turnover and by mitogen reactivity. Eur J Immunol. 1998;28(11):3738-48.
99. Schaniel C, Pardali E, Sallusto F, Speletas M, Ruedl C, Shimizu T, et al. Activated murine B lymphocytes and dendritic cells produce a novel CC chemokine which acts selectively on activated T cells. J Exp Med. 1998;188(3):451-63.
100. ten Boekel E, Melchers F, Rolink AG. Precursor B cells showing H chain allelic inclusion display allelic exclusion at the level of pre-B cell receptor surface expression. Immunity. 1998;8(2):199-207.
101. Ceredig R, Andersson J, Melchers F, Rolink A. Effect of deregulated IL-7 transgene expression on B lymphocyte development in mice expressing mutated pre-B cell receptors. Eur J Immunol. 1999;29(9):2797-807.
102. Ceredig R, Rolink AG, Melchers F, Andersson J. Fetal liver organ cultures as a tool to study selection processes during B cell development. Curr Top Microbiol Immunol. 1999;246:11-7; discussion 8-9.
103. Engel H, Rolink A, Weiss S. B cells are programmed to activate kappa and lambda for rearrangement at consecutive developmental stages. Eur J Immunol. 1999;29(7):2167-76.
104. Ghia P, Schaniel C, Rolink AG, Nadler LM, Cardoso AA. Human macrophage-derived chemokine (MDC) is strongly expressed following activation of both normal and malignant precursor and mature B cells. Curr Top Microbiol Immunol. 1999;246:103-10.
105. Melchers F, Rolink AG, Schaniel C. The role of chemokines in regulating cell migration during humoral immune responses. Cell. 1999;99(4):351-4.
106. Melchers F, ten Boekel E, Yamagami T, Andersson J, Rolink A. The roles of preB and B cell receptors in the stepwise allelic exclusion of mouse IgH and L chain gene loci. Semin Immunol. 1999;11(5):307-17.
107. Nutt SL, Heavey B, Rolink AG, Busslinger M. Commitment to the B-lymphoid lineage depends on the transcription factor Pax5. Nature. 1999;401(6753):556-62.
108. Nutt SL, Rolink AG, Busslinger M. The molecular basis of B-cell lineage commitment. Cold Spring Harb Symp Quant Biol. 1999;64:51-9.
109. Nutt SL, Vambrie S, Steinlein P, Kozmik Z, Rolink A, Weith A, et al. Independent regulation of the two Pax5 alleles during B-cell development. Nat Genet. 1999;21(4):390-5.
110. Rolink A, Nutt S, Busslinger M, ten Boekel E, Seidl T, Andersson J, et al. Differentiation, dedifferentiation, and redifferentiation of B-lineage lymphocytes: roles of the surrogate light chain and the Pax5 gene. Cold Spring Harb Symp Quant Biol. 1999;64:21-5.
111. Rolink AG, Brocker T, Bluethmann H, Kosco-Vilbois MH, Andersson J, Melchers F. Mutations affecting either generation or survival of cells influence the pool size of mature B cells. Immunity. 1999;10(5):619-28.
112. Rolink AG, Melchers F, Andersson J. The transition from immature to mature B cells. Curr Top Microbiol Immunol. 1999;246:39-43; discussion 4.
113. Rolink AG, Nutt SL, Melchers F, Busslinger M. Long-term in vivo reconstitution of T-cell development by Pax5-deficient B-cell progenitors. Nature. 1999;401(6753):603-6.
114. Rolink AG, ten Boekel E, Yamagami T, Ceredig R, Andersson J, Melchers F. B cell development in the mouse from early progenitors to mature B cells. Immunol Lett. 1999;68(1):89-93.
115. Schaniel C, Sallusto F, Ruedl C, Sideras P, Melchers F, Rolink AG. Three chemokines with potential functions in T lymphocyte-independent and -dependent B lymphocyte stimulation. Eur J Immunol. 1999;29(9):2934-47.
116. Schaniel C, Sallusto F, Sideras P, Melchers F, Rolink AG. A novel CC chemokine ABCD-1, produced by dendritic cells and activated B cells, exclusively attracts activated T lymphocytes. Curr Top Microbiol Immunol. 1999;246:95-101.
117. ten Boekel E, Yamagami T, Andersson J, Rolink AG, Melchers F. The formation and selection of cells expressing preB cell receptors and B cell receptors. Curr Top Microbiol Immunol. 1999;246:3-9; discussion -10.
118. Yamagami T, ten Boekel E, Andersson J, Rolink A, Melchers F. Frequencies of multiple IgL chain gene rearrangements in single normal or kappaL chain-deficient B lineage cells. Immunity. 1999;11(3):317-27.
119. Yamagami T, ten Boekel E, Schaniel C, Andersson J, Rolink A, Melchers F. Four of five RAG-expressing JCkappa-/- small pre-BII cells have no L chain gene rearrangements: detection by high-efficiency single cell PCR. Immunity. 1999;11(3):309-16.
120. Yu W, Nagaoka H, Jankovic M, Misulovin Z, Suh H, Rolink A, et al. Continued RAG expression in late stages of B cell development and no apparent re-induction after immunization. Nature. 1999;400(6745):682-7.
121. Busslinger M, Nutt SL, Rolink AG. Lineage commitment in lymphopoiesis. Curr Opin Immunol. 2000;12(2):151-8.
122. Ceredig R, Rolink AG, Melchers F, Andersson J. The B cell receptor, but not the pre-B cell receptor, mediates arrest of B cell differentiation. Eur J Immunol. 2000;30(3):759-67.
123. Ghia P, Melchers F, Rolink AG. Age-dependent changes in B lymphocyte development in man and mouse. Exp Gerontol. 2000;35(2):159-65.
124. Melchers F, ten Boekel E, Seidl T, Kong XC, Yamagami T, Onishi K, et al. Repertoire selection by pre-B-cell receptors and B-cell receptors, and genetic control of B-cell development from immature to mature B cells. Immunol Rev. 2000;175:33-46.
125. Rolink AG, Melchers F. Precursor B cells from Pax-5-deficient mice–stem cells for macrophages, granulocytes, osteoclasts, dendritic cells, natural killer cells, thymocytes and T cells. Curr Top Microbiol Immunol. 2000;251:21-6.
126. Rolink AG, Schaniel C, Busslinger M, Nutt SL, Melchers F. Fidelity and infidelity in commitment to B-lymphocyte lineage development. Immunol Rev. 2000;175:104-11.
127. Rolink AG, Winkler T, Melchers F, Andersson J. Precursor B cell receptor-dependent B cell proliferation and differentiation does not require the bone marrow or fetal liver environment. J Exp Med. 2000;191(1):23-32.
128. Schaniel C, Melchers F, Rolink AG. The cluster of ABCD chemokines which organizes T cell-dependent B cell responses. Curr Top Microbiol Immunol. 2000;251:181-9.
129. Schubart DB, Rolink A, Schubart K, Matthias P. Cutting edge: lack of peripheral B cells and severe agammaglobulinemia in mice simultaneously lacking Bruton's tyrosine kinase and the B cell-specific transcriptional coactivator OBF-1. J Immunol. 2000;164(1):18-22.
130. Ghia P, Transidico P, Veiga JP, Schaniel C, Sallusto F, Matsushima K, et al. Chemoattractants MDC and TARC are secreted by malignant B-cell precursors following CD40 ligation and support the migration of leukemia-specific T cells. Blood. 2001;98(3):533-40.
131. Nutt SL, Eberhard D, Horcher M, Rolink AG, Busslinger M. Pax5 determines the identity of B cells from the beginning to the end of B-lymphopoiesis. Int Rev Immunol. 2001;20(1):65-82.
132. Rolink AG, Schaniel C, Andersson J, Melchers F. Selection events operating at various stages in B cell development. Curr Opin Immunol. 2001;13(2):202-7.
133. Schaniel C, Rolink AG, Melchers F. Attractions and migrations of lymphoid cells in the organization of humoral immune responses. Adv Immunol. 2001;78:111-68.
134. Schubart K, Massa S, Schubart D, Corcoran LM, Rolink AG, Matthias P. B cell development and immunoglobulin gene transcription in the absence of Oct-2 and OBF-1. Nat Immunol. 2001;2(1):69-74.
135. Seidl T, Rolink A, Melchers F. The VpreB protein of the surrogate light-chain can pair with some mu heavy-chains in the absence of the lambda 5 protein. Eur J Immunol. 2001;31(7):1999-2006.
136. Terszowski G, Jankowski A, Hendriks WJ, Rolink AG, Kisielow P. Within the hemopoietic system, LAR phosphatase is a T cell lineage-specific adhesion receptor-like protein whose phosphatase activity appears dispensable for T cell development, repertoire selection and function. Eur J Immunol. 2001;31(3):832-40.
137. Bruno L, Schaniel C, Rolink A. Plasticity of Pax-5(-/-) pre-B I cells. Cells Tissues Organs. 2002;171(1):38-43.
138. Hoffmann R, Seidl T, Neeb M, Rolink A, Melchers F. Changes in gene expression profiles in developing B cells of murine bone marrow. Genome Res. 2002;12(1):98-111.
139. Martensson IL, Rolink A, Melchers F, Mundt C, Licence S, Shimizu T. The pre-B cell receptor and its role in proliferation and Ig heavy chain allelic exclusion. Semin Immunol. 2002;14(5):335-42.
140. Rolink AG, Melchers F. BAFFled B cells survive and thrive: roles of BAFF in B-cell development. Curr Opin Immunol. 2002;14(2):266-75.
141. Rolink AG, Schaniel C, Bruno L, Melchers F. In vitro and in vivo plasticity of Pax5-deficient pre-B I cells. Immunol Lett. 2002;82(1-2):35-40.
142. Rolink AG, Schaniel C, Melchers F. Stability and plasticity of wild-type and Pax5-deficient precursor B cells. Immunol Rev. 2002;187:87-95.
143. Rolink AG, Tschopp J, Schneider P, Melchers F. BAFF is a survival and maturation factor for mouse B cells. Eur J Immunol. 2002;32(7):2004-10.
144. Schaniel C, Bruno L, Melchers F, Rolink AG. Multiple hematopoietic cell lineages develop in vivo from transplanted Pax5-deficient pre-B I-cell clones. Blood. 2002;99(2):472-8.
145. Schaniel C, Gottar M, Roosnek E, Melchers F, Rolink AG. Extensive in vivo self-renewal, long-term reconstitution capacity, and hematopoietic multipotency of Pax5-deficient precursor B-cell clones. Blood. 2002;99(8):2760-6.
146. Ceredig R, Bosco N, Maye PN, Andersson J, Rolink A. In interleukin-7-transgenic mice, increasing B lymphopoiesis increases follicular but not marginal zone B cell numbers. Eur J Immunol. 2003;33(9):2567-76.
147. Duber S, Engel H, Rolink A, Kretschmer K, Weiss S. Germline transcripts of immunoglobulin light chain variable regions are structurally diverse and differentially expressed. Mol Immunol. 2003;40(8):509-16.
148. Felix K, Rolink A, Melchers F, Janz S. Bcl-2 reduces mutant rates in a transgenic lacZ reporter gene in mouse pre-B lymphocytes. Mutat Res. 2003;522(1-2):135-44.
149. Hoffmann R, Bruno L, Seidl T, Rolink A, Melchers F. Rules for gene usage inferred from a comparison of large-scale gene expression profiles of T and B lymphocyte development. J Immunol. 2003;170(3):1339-53.
150. Rolink AG. B-cell development and pre-B-1 cell plasticity in vitro. Methods Mol Biol. 2004;271:271-81.
151. Rolink AG, Andersson J, Melchers F. Molecular mechanisms guiding late stages of B-cell development. Immunol Rev. 2004;197:41-50.
152. Tardivel A, Tinel A, Lens S, Steiner QG, Sauberli E, Wilson A, et al. The anti-apoptotic factor Bcl-2 can functionally substitute for the B cell survival but not for the marginal zone B cell differentiation activity of BAFF. Eur J Immunol. 2004;34(2):509-18.
153. Ardouin L, Rolink AG, Mura AM, Gommeaux J, Melchers F, Busslinger M, et al. Rapid in vivo analysis of mutant forms of the LAT adaptor using Pax5-Lat double-deficient pro-B cells. Eur J Immunol. 2005;35(3):977-86.
154. Balciunaite G, Ceredig R, Fehling HJ, Zuniga-Pflucker JC, Rolink AG. The role of Notch and IL-7 signaling in early thymocyte proliferation and differentiation. Eur J Immunol. 2005;35(4):1292-300.
155. Balciunaite G, Ceredig R, Massa S, Rolink AG. A B220+ CD117+ CD19- hematopoietic progenitor with potent lymphoid and myeloid developmental potential. Eur J Immunol. 2005;35(7):2019-30.
156. Balciunaite G, Ceredig R, Rolink AG. The earliest subpopulation of mouse thymocytes contains potent T, significant macrophage, and natural killer cell but no B-lymphocyte potential. Blood. 2005;105(5):1930-6.
157. Harfst E, Andersson J, Grawunder U, Ceredig R, Rolink AG. Homeostatic and functional analysis of mature B cells in lambda5-deficient mice. Immunol Lett. 2005;101(2):173-84.
158. Matthias P, Rolink AG. Transcriptional networks in developing and mature B cells. Nat Rev Immunol. 2005;5(6):497-508.
159. Benard A, Ceredig R, Rolink AG. Regulatory T cells control autoimmunity following syngeneic bone marrow transplantation. Eur J Immunol. 2006;36(9):2324-35.
160. Bosco N, Agenes F, Rolink AG, Ceredig R. Peripheral T cell lymphopenia and concomitant enrichment in naturally arising regulatory T cells: the case of the pre-Talpha gene-deleted mouse. J Immunol. 2006;177(8):5014-23.
161. Ceredig R, Rauch M, Balciunaite G, Rolink AG. Increasing Flt3L availability alters composition of a novel bone marrow lymphoid progenitor compartment. Blood. 2006;108(4):1216-22.
162. Maerki S, Ceredig R, Rolink A. Induction of chemokine receptor expression during early stages of T cell development. Immunol Lett. 2006;104(1-2):110-7.
163. Massa S, Balciunaite G, Ceredig R, Rolink AG. Critical role for c-kit (CD117) in T cell lineage commitment and early thymocyte development in vitro. Eur J Immunol. 2006;36(3):526-32.
164. Melchers F, Rolink AR. B cell tolerance–how to make it and how to break it. Curr Top Microbiol Immunol. 2006;305:1-23.
165. Rolink AG, Balciunaite G, Demoliere C, Ceredig R. The potential involvement of Notch signaling in NK cell development. Immunol Lett. 2006;107(1):50-7.
166. Rolink AG, Massa S, Balciunaite G, Ceredig R. Early lymphocyte development in bone marrow and thymus. Swiss Med Wkly. 2006;136(43-44):679-83.
167. Aschenbrenner K, D'Cruz LM, Vollmann EH, Hinterberger M, Emmerich J, Swee LK, et al. Selection of Foxp3+ regulatory T cells specific for self antigen expressed and presented by Aire+ medullary thymic epithelial cells. Nat Immunol. 2007;8(4):351-8.
168. Brown G, Hughes PJ, Michell RH, Rolink AG, Ceredig R. The sequential determination model of hematopoiesis. Trends Immunol. 2007;28(10):442-8.
169. Ceredig R, Bosco N, Rolink AG. The B lineage potential of thymus settling progenitors is critically dependent on mouse age. Eur J Immunol. 2007;37(3):830-7.
170. Chappaz S, Flueck L, Farr AG, Rolink AG, Finke D. Increased TSLP availability restores T- and B-cell compartments in adult IL-7 deficient mice. Blood. 2007;110(12):3862-70.
171. Hoffmann R, Lottaz C, Kuhne T, Rolink A, Melchers F. Neutrality, compensation, and negative selection during evolution of B-cell development transcriptomes. Mol Biol Evol. 2007;24(12):2610-8.
172. Melchers F, Yamagami T, Rolink A, Andersson J. Rules for the rearrangement events at the L chain gene loci of the mouse. Adv Exp Med Biol. 2007;596:63-70.
173. Rolink AG, Massa S, Balciunaite G, Ceredig R. Early lymphocyte development in bone marrow and thymus. Swiss Med Wkly. 2007;137 Suppl 155:20S-4S.
174. Bordon A, Bosco N, Du Roure C, Bartholdy B, Kohler H, Matthias G, et al. Enforced expression of the transcriptional coactivator OBF1 impairs B cell differentiation at the earliest stage of development. PLoS One. 2008;3(12):e4007.
175. Bosco N, Ceredig R, Rolink A. Transient decrease in interleukin-7 availability arrests B lymphopoiesis during pregnancy. Eur J Immunol. 2008;38(2):381-90.
176. Bosco N, Engdahl C, Benard A, Rolink J, Ceredig R, Rolink AG. TCR-beta chains derived from peripheral gammadelta T cells can take part in alphabeta T-cell development. Eur J Immunol. 2008;38(12):3520-9.
177. Fazio G, Palmi C, Rolink A, Biondi A, Cazzaniga G. PAX5/TEL acts as a transcriptional repressor causing down-modulation of CD19, enhances migration to CXCL12, and confers survival advantage in pre-BI cells. Cancer Res. 2008;68(1):181-9.
178. Mueller P, Massner J, Jayachandran R, Combaluzier B, Albrecht I, Gatfield J, et al. Regulation of T cell survival through coronin-1-mediated generation of inositol-1,4,5-trisphosphate and calcium mobilization after T cell receptor triggering. Nat Immunol. 2008;9(4):424-31.
179. Tiao JY, Bradaia A, Biermann B, Kaupmann K, Metz M, Haller C, et al. The sushi domains of secreted GABA(B1) isoforms selectively impair GABA(B) heteroreceptor function. J Biol Chem. 2008;283(45):31005-11.
180. Ceredig R, Rolink AG, Brown G. Models of haematopoiesis: seeing the wood for the trees. Nat Rev Immunol. 2009;9(4):293-300.
181. Rauch M, Tussiwand R, Bosco N, Rolink AG. Crucial role for BAFF-BAFF-R signaling in the survival and maintenance of mature B cells. PLoS One. 2009;4(5):e5456.
182. Schmutz S, Bosco N, Chappaz S, Boyman O, Acha-Orbea H, Ceredig R, et al. Cutting edge: IL-7 regulates the peripheral pool of adult ROR gamma+ lymphoid tissue inducer cells. J Immunol. 2009;183(4):2217-21.
183. Swee LK, Bosco N, Malissen B, Ceredig R, Rolink A. Expansion of peripheral naturally occurring T regulatory cells by Fms-like tyrosine kinase 3 ligand treatment. Blood. 2009;113(25):6277-87.
184. Tussiwand R, Bosco N, Ceredig R, Rolink AG. Tolerance checkpoints in B-cell development: Johnny B good. Eur J Immunol. 2009;39(9):2317-24.
185. Bosco N, Swee LK, Benard A, Ceredig R, Rolink A. Auto-reconstitution of the T-cell compartment by radioresistant hematopoietic cells following lethal irradiation and bone marrow transplantation. Exp Hematol. 2010;38(3):222-32 e2.
186. Cassani B, Poliani PL, Marrella V, Schena F, Sauer AV, Ravanini M, et al. Homeostatic expansion of autoreactive immunoglobulin-secreting cells in the Rag2 mouse model of Omenn syndrome. J Exp Med. 2010;207(7):1525-40.
187. Santiago-Raber ML, Amano H, Amano E, Fossati-Jimack L, Swee LK, Rolink A, et al. Evidence that Yaa-induced loss of marginal zone B cells is a result of dendritic cell-mediated enhanced activation. J Autoimmun. 2010;34(4):349-55.
188. Swee LK, Tardivel A, Schneider P, Rolink A. Rescue of the mature B cell compartment in BAFF-deficient mice by treatment with recombinant Fc-BAFF. Immunol Lett. 2010;131(1):40-8.
189. Bossen C, Tardivel A, Willen L, Fletcher CA, Perroud M, Beermann F, et al. Mutation of the BAFF furin cleavage site impairs B-cell homeostasis and antibody responses. Eur J Immunol. 2011;41(3):787-97.
190. Tussiwand R, Engdahl C, Gehre N, Bosco N, Ceredig R, Rolink AG. The preTCR-dependent DN3 to DP transition requires Notch signaling, is improved by CXCL12 signaling and is inhibited by IL-7 signaling. Eur J Immunol. 2011;41(11):3371-80.
191. Ceredig R, Rolink AG. The key role of IL-7 in lymphopoiesis. Semin Immunol. 2012;24(3):159-64.
192. Kreuzaler M, Rauch M, Salzer U, Birmelin J, Rizzi M, Grimbacher B, et al. Soluble BAFF levels inversely correlate with peripheral B cell numbers and the expression of BAFF receptors. J Immunol. 2012;188(1):497-503.
193. Tussiwand R, Rauch M, Fluck LA, Rolink AG. BAFF-R expression correlates with positive selection of immature B cells. Eur J Immunol. 2012;42(1):206-16.
194. Fazio G, Cazzaniga V, Palmi C, Galbiati M, Giordan M, te Kronnie G, et al. PAX5/ETV6 alters the gene expression profile of precursor B cells with opposite dominant effect on endogenous PAX5. Leukemia. 2013;27(4):992-5.
195. Kyaw T, Cui P, Tay C, Kanellakis P, Hosseini H, Liu E, et al. BAFF receptor mAb treatment ameliorates development and progression of atherosclerosis in hyperlipidemic ApoE(-/-) mice. PLoS One. 2013;8(4):e60430.
196. Kraus H, Kaiser S, Aumann K, Bonelt P, Salzer U, Vestweber D, et al. A feeder-free differentiation system identifies autonomously proliferating B cell precursors in human bone marrow. J Immunol. 2014;192(3):1044-54.
197. Nusser A, Nuber N, Wirz OF, Rolink H, Andersson J, Rolink A. The development of autoimmune features in aging mice is closely associated with alterations of the peripheral CD4(+) T-cell compartment. Eur J Immunol. 2014;44(10):2893-902.
198. Okujava R, Guye P, Lu YY, Mistl C, Polus F, Vayssier-Taussat M, et al. A translocated effector required for Bartonella dissemination from derma to blood safeguards migratory host cells from damage by co-translocated effectors. PLoS Pathog. 2014;10(6):e1004187.
199. Pieper K, Rizzi M, Speletas M, Smulski CR, Sic H, Kraus H, et al. A common single nucleotide polymorphism impairs B-cell activating factor receptor's multimerization, contributing to common variable immunodeficiency. J Allergy Clin Immunol. 2014;133(4):1222-5.
200. Swee LK, Nusser A, Curti M, Kreuzaler M, Rolink H, Terracciano L, et al. The amount of self-antigen determines the effector function of murine T cells escaping negative selection. Eur J Immunol. 2014;44(5):1299-312.
201. Tsapogas P, Swee LK, Nusser A, Nuber N, Kreuzaler M, Capoferri G, et al. In vivo evidence for an instructive role of fms-like tyrosine kinase-3 (FLT3) ligand in hematopoietic development. Haematologica. 2014;99(4):638-46.
202. Venhoff N, Niessen L, Kreuzaler M, Rolink AG, Hassler F, Rizzi M, et al. Reconstitution of the peripheral B lymphocyte compartment in patients with ANCA-associated vasculitides treated with rituximab for relapsing or refractory disease. Autoimmunity. 2014;47(6):401-8.
203. Vigano MA, Ivanek R, Balwierz P, Berninger P, van Nimwegen E, Karjalainen K, et al. An epigenetic profile of early T-cell development from multipotent progenitors to committed T-cell descendants. Eur J Immunol. 2014;44(4):1181-93.
204. von Burg N, Chappaz S, Baerenwaldt A, Horvath E, Bose Dasgupta S, Ashok D, et al. Activated group 3 innate lymphoid cells promote T-cell-mediated immune responses. Proc Natl Acad Sci U S A. 2014;111(35):12835-40.
205. von Muenchow L, Engdahl C, Karjalainen K, Rolink AG. The selection of mature B cells is critically dependent on the expression level of the co-receptor CD19. Immunol Lett. 2014;160(2):113-9.
206. Bessa J, Boeckle S, Beck H, Buckel T, Schlicht S, Ebeling M, et al. The immunogenicity of antibody aggregates in a novel transgenic mouse model. Pharm Res. 2015;32(7):2344-59.
207. Bornancin F, Renner F, Touil R, Sic H, Kolb Y, Touil-Allaoui I, et al. Deficiency of MALT1 paracaspase activity results in unbalanced regulatory and effector T and B cell responses leading to multiorgan inflammation. J Immunol. 2015;194(8):3723-34.
208. Brown G, Mooney CJ, Alberti-Servera L, Muenchow L, Toellner KM, Ceredig R, et al. Versatility of stem and progenitor cells and the instructive actions of cytokines on hematopoiesis. Crit Rev Clin Lab Sci. 2015;52(4):168-79.
209. Choukrallah MA, Song S, Rolink AG, Burger L, Matthias P. Enhancer repertoires are reshaped independently of early priming and heterochromatin dynamics during B cell differentiation. Nat Commun. 2015;6:8324.
210. Gehre N, Nusser A, von Muenchow L, Tussiwand R, Engdahl C, Capoferri G, et al. A stromal cell free culture system generates mouse pro-T cells that can reconstitute T-cell compartments in vivo. Eur J Immunol. 2015;45(3):932-42.
211. Harmeier A, Obermueller S, Meyer CA, Revel FG, Buchy D, Chaboz S, et al. Trace amine-associated receptor 1 activation silences GSK3beta signaling of TAAR1 and D2R heteromers. Eur Neuropsychopharmacol. 2015;25(11):2049-61.
212. Manoharan A, Du Roure C, Rolink AG, Matthias P. De novo DNA Methyltransferases Dnmt3a and Dnmt3b regulate the onset of Igkappa light chain rearrangement during early B-cell development. Eur J Immunol. 2015;45(8):2343-55.
213. Nobs SP, Schneider C, Dietrich MG, Brocker T, Rolink A, Hirsch E, et al. PI3-Kinase-gamma Has a Distinct and Essential Role in Lung-Specific Dendritic Cell Development. Immunity. 2015;43(4):674-89.
214. Baerenwaldt A, von Burg N, Kreuzaler M, Sitte S, Horvath E, Peter A, et al. Flt3 Ligand Regulates the Development of Innate Lymphoid Cells in Fetal and Adult Mice. J Immunol. 2016;196(6):2561-71.
215. von Muenchow L, Alberti-Servera L, Klein F, Capoferri G, Finke D, Ceredig R, et al. Permissive roles of cytokines interleukin-7 and Flt3 ligand in mouse B-cell lineage commitment. Proc Natl Acad Sci U S A. 2016;113(50):E8122-E30.
216. Alberti-Servera L, von Muenchow L, Tsapogas P, Capoferri G, Eschbach K, Beisel C, et al. Single-cell RNA sequencing reveals developmental heterogeneity among early lymphoid progenitors. EMBO J. 2017;36(24):3619-33.
217. Fazio G, Turazzi N, Cazzaniga V, Kreuzaler M, Maglia O, Magnani CF, et al. TNFRSF13C (BAFFR) positive blasts persist after early treatment and at relapse in childhood B-cell precursor acute lymphoblastic leukaemia. Br J Haematol. 2017.
218. Smulski CR, Kury P, Seidel LM, Staiger HS, Edinger AK, Willen L, et al. BAFF- and TACI-Dependent Processing of BAFFR by ADAM Proteases Regulates the Survival of B Cells. Cell Rep. 2017;18(9):2189-202.
219. Tsapogas P, Mooney CJ, Brown G, Rolink A. The Cytokine Flt3-Ligand in Normal and Malignant Hematopoiesis. Int J Mol Sci. 2017;18(6).
220. von Muenchow L, Tsapogas P, Alberti-Servera L, Capoferri G, Doelz M, Rolink H, et al. Pro-B cells propagated in stromal cell-free cultures reconstitute functional B-cell compartments in immunodeficient mice. Eur J Immunol. 2017;47(2):394-405.
221. Calvo-Asensio I, Sugrue T, Bosco N, Rolink A, Ceredig R. DN2 Thymocytes Activate a Specific Robust DNA Damage Response to Ionizing Radiation-Induced DNA Double-Strand Breaks. Front Immunol. 2018;9:1312.
222. Faderl M, Klein F, Wirz OF, Heiler S, Alberti-Servera L, Engdahl C, et al. Two Distinct Pathways in Mice Generate Antinuclear Antigen-Reactive B Cell Repertoires. Front Immunol. 2018;9:16.
223. Heiler S, Lotscher J, Kreuzaler M, Rolink J, Rolink A. Prophylactic and Therapeutic Effects of Interleukin-2 (IL-2)/Anti-IL-2 Complexes in Systemic Lupus Erythematosus-Like Chronic Graft-Versus-Host Disease. Front Immunol. 2018;9:656.
224. Kim M, von Muenchow L, Le Meur T, Kueng B, Gapp B, Weber D, et al. DPP9 enzymatic activity in hematopoietic cells is dispensable for mouse hematopoiesis. Immunol Lett. 2018;198:60-5.
225. Turazzi N, Fazio G, Rossi V, Rolink A, Cazzaniga G, Biondi A, et al. Engineered T cells towards TNFRSF13C (BAFFR): a novel strategy to efficiently target B-cell acute lymphoblastic leukaemia. Br J Haematol. 2018.
226. Vigolo M, Chambers MG, Willen L, Chevalley D, Maskos K, Lammens A, et al. A loop region of BAFF controls B cell survival and regulates recognition by different inhibitors. Nat Commun. 2018;9(1):1199.
227. Wilhelmson AS, Lantero Rodriguez M, Stubelius A, Fogelstrand P, Johansson I, Buechler MB, et al. Testosterone is an endogenous regulator of BAFF and splenic B cell number. Nat Commun. 2018;9(1):2067.
